# Genome Sequences of Two Methicillin-Sensitive Staphylococcus aureus Healthy Skin Isolates

**DOI:** 10.1128/mra.00402-22

**Published:** 2022-05-16

**Authors:** Krista B. Mills, Fan Jia, Michelle E. Stein, Klara C. Keim, Rebecca M. Davidson, Alexander R. Horswill

**Affiliations:** a Department of Immunology and Microbiology, University of Colorado Anschutz Medical Campus, Aurora, Colorado, USA; b Center for Genes, Environment, and Health, Department of Immunology and Genomic Medicine, National Jewish Health, Denver, Colorado, USA; University of Rochester School of Medicine and Dentistry

## Abstract

The USA300 and USA600 clonal lineages are the cause of many serious Staphylococcus aureus infections. Here, we report the complete genomes of two methicillin-sensitive S. aureus strains isolated from the healthy skin of adults in Colorado, which are most phylogenetically similar to the USA300 and USA600 lineages.

## ANNOUNCEMENT

Staphylococcus aureus causes the majority of all skin and soft tissue infections (1, 2) but colonizes healthy skin at only low rates (3). A better understanding of colonization determinants could provide insight into how S. aureus transitions into infection. To address this knowledge gap, we present the complete genomes of two methicillin-sensitive S. aureus isolates from the healthy skin of adults in Colorado.

AH5611 and AH5667 were obtained from healthy dry skin (dorsal forearm) and moist skin (antecubital fossa), respectively, of an adult by swabbing (Colorado Multiple Institutional Review Board [COMIRB]-approved protocol 19-2218). The strains were grown in tryptic soy broth (TSB) for 16 h at 37°C. Cells were lysed with lysostaphin and lysozyme, followed by purification of whole genomes using the DNeasy blood and tissue kit (Qiagen, Hilden, Germany). Whole-genome sequences were acquired using 2 × 150-bp reads from a NextSeq 2000 sequencer (Illumina, Inc., San Diego, CA) and long reads from a MinION sequencer (Oxford Nanopore Technologies [ONT], Oxford, UK). Libraries were prepared using the Illumina DNA preparation kit, and adapter trimming of reads was performed with bcl2fastq v2.20.0.445 (4). ONT libraries were prepared with the SQK-LSK109 ligation sequencing kit (ONT) and sequenced with the R9.4 MinION flow cell. Quality control and base calling of ONT reads were performed with GuPPy v5.0.16 high accuracy mode (HAC) (5), and adapter trimming was performed with Porechop v0.2.3 (6). ONT reads had *N*_50_ values of 20,934 bp and 18,834 bp for AH5611 and AH5667, respectively. Hybrid assemblies of Illumina and ONT reads were performed with Unicycler v0.4.8 (7), and genome annotations were performed with Prokka v1.13.3 (8). Default parameters were used for all software.

The complete genome of AH5611 was assembled using 3,582,864 Illumina read pairs (mean coverage of 349×) and 51,180 ONT reads (mean coverage of 286×). AH5611 has a 2,700,946-bp circular chromosome, with a G+C content of 32.94%, and was identified as sequence type 45 (ST45), clonal complex 45 (CC45), and *agr* type I. AH5611 was most phylogenetically similar to CA-347 (a USA600 strain, GenBank accession no. CP006044) (9). Genome annotation identified 2,479 coding sequences (CDSs), 59 tRNAs, and 19 rRNAs. PHASTER analysis (10) revealed three incomplete prophages.

The complete genome and two plasmids of AH5667 were assembled with 3,209,571 Illumina read pairs (mean coverage of 307×) and 52,309 ONT reads (mean coverage of 239×). AH5667 has a 2,817,030-bp circular chromosome, with a G+C content of 32.81%, and was identified as ST8, CC8, and *agr* type I. AH5667 was most phylogenetically similar to FPR3757 (a USA300 strain, GenBank accession no. CP000255) (11). AH5667 has 2,595 CDSs, 62 tRNAs, and 19 rRNAs. pAH5661_1 is 13,162 bp, with a G+C content of 28.08%, and pAH5667_2 is 3,125 bp, with a G+C content of 28.74%; they are homologous to pUSA04-1 and pUSA01-1, respectively (11). PHASTER analysis revealed two complete prophages in the chromosome, which are most similar to phi2958PVL (GenBank accession no. NC_011344.1) and 23MRA (GenBank accession no. NC_028775.1).

The genomes of AH5611 and AH5667 most closely resemble the USA600 and USA300 clonal lineages, respectively. These clonal lineages are the cause of many life-threatening infections (12–14). Future analyses may provide insight into determinants of S. aureus healthy skin colonization.

### Data availability.

The complete sequences of the chromosomes of AH5611 and AH5667 have been deposited in GenBank under accession no. CP092055 (BioSample accession no. SAMN25144898) and accession no. CP092052 (BioSample accession no. SAMN25144899), respectively. The complete sequence of AH5667 plasmid pAH5667_1 has been deposited in GenBank under accession no. CP092053 (BioSample accession no. SAMN25144899). The complete sequence of AH5667 plasmid pAH5667_2 has been deposited in GenBank under accession no. CP092054 (BioSample accession no. SAMN25144899). SRA reads are available under BioProject accession no. PRJNA799058.
